# Bacterial Cell Morphogenesis Does Not Require a Preexisting Template Structure

**DOI:** 10.1016/j.cub.2014.02.053

**Published:** 2014-04-14

**Authors:** Yoshikazu Kawai, Romain Mercier, Jeff Errington

**Affiliations:** 1 Centre for Bacterial Cell Biology, Institute for Cell and Molecular Biosciences, Medical School, Newcastle University, Richardson Road, Newcastle upon Tyne NE2 4AX, UK

## Abstract

Morphogenesis, the development of shape or form in cells or organisms, is a fundamental but poorly understood process throughout biology. In the bacterial domain, cells have a wide range of characteristic shapes, including rods, cocci, and spirals. The cell wall, composed of a simple meshwork of long glycan strands crosslinked by short peptides (peptidoglycan, PG) and anionic cell wall polymers such as wall teichoic acids (WTAs), is the major determinant of cell shape. It has long been debated whether the formation of new wall material or the transmission of shape from parent to daughter cells requires existing wall material as a template [1–3]. However, rigorous testing of this hypothesis has been problematical because the cell wall is normally an essential structure. L-forms are wall-deficient variants of common bacteria that have been classically identified as antibiotic-resistant variants in association with a wide range of infectious diseases [4–6]. We recently determined the genetic basis for the L-form transition in the rod-shaped bacterium *Bacillus subtilis* and thus how to generate L-forms reliably and reproducibly [7, 8]. Using the new L-form system, we show here that we can delete essential genes for cell wall synthesis and propagate cells in the long-term absence of a cell wall template molecule. Following genetic restoration of cell wall synthesis, we show that the ability to generate a classical rod-shaped cell is restored, conclusively rejecting template-directed models, at least for the establishment of cell shape in *B. subtilis*.

## Highlights

• Essential cell wall synthetic genes can be deleted in *B. subtilis* L-forms
• Reintroduction of the genes restores cell wall synthesis
• This is sufficient to regenerate the cell wall and correct cell morphology
• Cell morphogenesis does not require a preexisting cell wall template

## Teaser

The cell wall is thought to specify cell shape in bacteria. Kawai et al. have taken advantage of L-form bacteria to delete genes essential for wall synthesis. They then developed a system to restore cell wall synthesis and show that cell shape can be regenerated. This work conclusively excludes template-dependent models for cell morphogenesis.

## Results and Discussion

**Figure 1:**
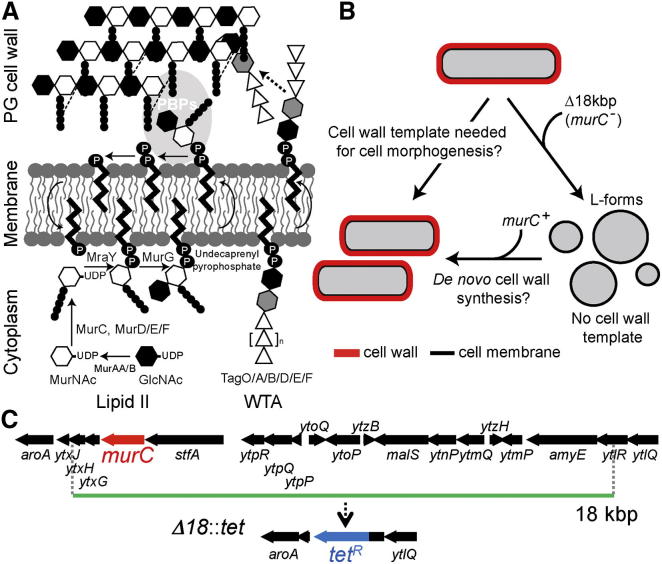
Schematic View of Peptidoglycan Synthesis and the Models for Cell Morphogenesis (A) The peptidoglycan (PG) cell wall is built from long glycan strands composed of N-acetylmuramic acid (MurNAc) and N-acetylglucosamine (GlcNAc) crosslinked by peptide cross-bridges [10, 11]. The precursor for PG is initially synthesized in the cytoplasm by the action of MurAA, MurB, MurC, MurD, MurE, and MurF enzymes. MurNAc-pentapeptide is coupled to a membrane carrier, undecaprenyl pyrophosphate, by MraY, and GlcNAc is added by MurG to form lipid II, which is then transferred to the outside of the cytoplasmic membrane. Newly synthesized PG is incorporated into the existing PG meshwork by a combination of transglycosylation and transpeptidation reactions catalyzed by penicillin-binding proteins. Wall teichoic acids (WTAs) are abundant PG-linked glycopolymers present in most Gram-positive organisms and are essential for maintaining rod shape in *B. subtilis* [12]. WTA synthesis begins at the cytoplasmic side of membrane with the coupling of GlcNAc to the same lipid carrier, undecaprenyl pyrophospate, as is used for PG precursors. WTA polymer synthesis requires the action of series of enzymes (TagA, TagB, TagD, TagE, and TgaF) in the cytosol. The polymer is exported and coupled to the PG by the action of the TagTUV enzymes [13]. (B) Models for bacterial cell morphogenesis. It has not been clear whether the formation of new wall material and the transmission of rod shape from parent to daughter cells require existing wall material as a template (see text). (C) Schematic representation of the chromosomal region deleted in Δ*18*::*tet* L-forms [8].

It is well known that treatment of bacterial cells with cell wall-active antibiotics or enzymes such as lysozymes converts them to cell wall-deficient protoplasts, which can be maintained in an osmoprotective medium (though with little net growth [8]). Such protoplasts can then be regenerated to produce viable, walled cells with normal morphology [2], albeit at low efficiency (reviewed by Hopwood [9]). In these experiments, it is difficult to exclude the presence of residual cell wall template fragments because synthesis and assembly of the wall can continue via newly synthesized precursors or catalytic enzymes (Figure 1A) [10–13]. However, bacterial variants called L-forms [5] are capable of prolonged growth in the absence of cell wall synthesis and thus might be suited to a definitive test of the need for a cell wall template. Because they are largely or completely lacking in cell wall, the basic shape of L-forms is spherical, but they are highly malleable and take on an array of irregular shapes influenced by the surrounding milieu. L-forms have often been classified as “stable,” in which case the cells can be propagated in the L-form state indefinitely, or as “unstable” for strains capable of reverting to the normal walled state. In principle, the existence of unstable L-forms suggests that a defined cell shape can be generated de novo. However, recent work on *Escherichia coli* L-forms suggests that unstable L-forms retain the requirement for at least a low level of cell wall synthesis, because genes essential for cell wall synthesis or assembly remain essential in the unstable L-forms [14, 15].

We have been developing methods for generating L-forms of the Gram-positive model bacterium *Bacillus subtilis* [7, 8, 16]. We found that at least two mutations are normally required for L-form growth. One mutation (e.g., *ispA*) has a poorly defined role in maintaining cell integrity and is probably of little direct functional significance. The key mutations enabling proliferation in the L-form state appear to work simply by increasing the rate of membrane synthesis. L-forms proliferate by a strange mechanism of membrane blebbing, or tubulation and fission [7]; it seems that excess membrane synthesis is sufficient to drive this mode of cell division [8]. Genetic screens revealed two classes of mutation that can generate the excess membrane effect. One class (i.e., overexpression of the gene encoding the catalytic subunit of acetyl-coenzyme A-carboxylase [AccDA] [8]) leads directly to upregulation of the fatty acid synthetic pathway and hence to increased membrane synthesis. The other class, inhibition of cell wall precursor synthesis (e.g., by repression of the *murE* operon [7]), works indirectly by an as yet uncharacterized mechanism. Nevertheless, the fact that repression of peptidoglycan (PG) precursor synthesis can promote the L-form transition provides a means, in principle, of testing whether continued PG synthesis is needed to maintain the ability to regenerate a rod-shaped walled cell (Figure 1B).

**Figure 2:**
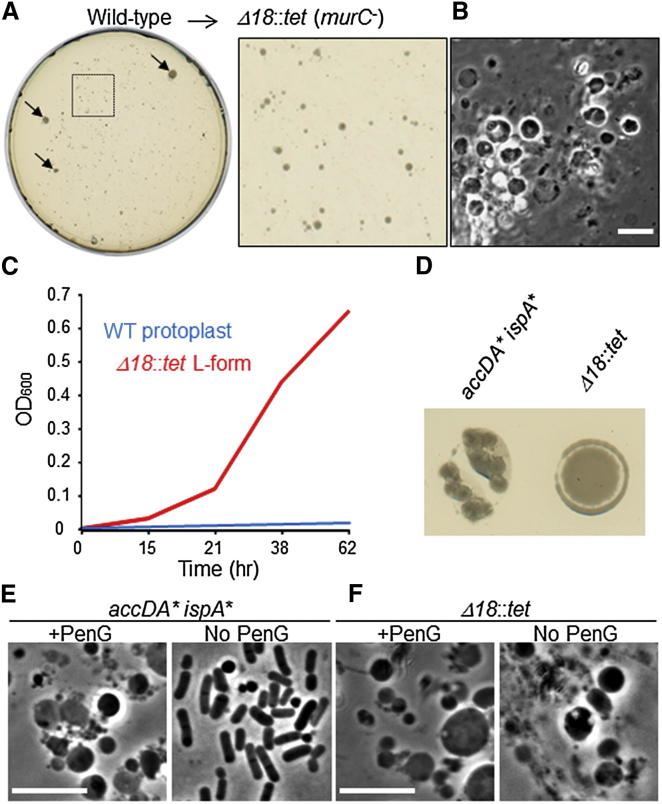
Inhibition of PG Precursor Synthesis by Deleting *murC* (A) Transformation of wild-type walled cells to L-forms by introduction of the Δ*18*::*tet* mutation, which completely deletes the *murC* gene as well as 17 other genes. Colonies of Δ*18*::*tet* L-forms were selected on transformation plates (NA/MSM) containing tetracycline and benzamide. The plate was incubated for 4 days at 30°C after transformation. An enlarged image of the typical colonies (dashed square) is shown to the right. Arrows point to large dense colonies that were of spurious origin and contained walled rod-shaped cells. (B) Phase-contrast micrograph of Δ*18*::*tet* L-forms from a colony on the transformation plate shown in (A). Scale bar represents 5 μm. (C) Growth of wild-type protoplasts (WT, blue) and Δ*18*::*tet* L-forms (Δ*18*::*tet*, red) in L-form-supporting medium (NB/MSM) with benzamide. Cells were incubated at 30°C. (D) Induction of cell wall regeneration from L-forms of strain RM84 [8] (*accDA*^∗^*ispA*^∗^) or Δ*18*::*tet*. Proliferating L-form cultures (OD_600_ = ∼0.2–0.3) in L-form medium (NB/MSM) with penicillin G (PenG) and benzamide were spotted onto L-form plates (NA/MSM) without PenG and benzamide. The plates were incubated for 3 days at 30°C. (E) Phase-contrast micrograph of proliferating L-forms of strain RM84 (*accDA*^∗^*ispA*^∗^) in L-form medium (NB/MSM) with penicillin G and benzamide (left), and after induction of cell wall regeneration by cultivating L-forms (left) on L-form plates (NA/MSM) in the absence of PenG and benzamide (right). Phase-contrast micrograph of rod-shaped cells was taken from a colony shown in (D) (*accDA*^∗^*ispA*^∗^). Scale bar represents 5 μm. (F) Phase-contrast micrograph of proliferating L-forms of Δ*18*::*tet* in L-form medium with penicillin G and benzamide (left) or from the L-form regeneration plate (no penicillin G or benzamide) shown in (D) (Δ*18*::*tet*) (right). Scale bar represents 5 μm.

In our previous work, we identified an 18 kbp deletion that enables stable proliferation of L-forms [8]. This deletion removed the *murC* gene, which encodes an essential enzyme in the PG precursor pathway, together with 17 other coding regions of mainly unknown function. (We assume that one or more of the other genes deleted confer a stabilizing effect similar to that of the *ispA* mutation mentioned above, although we have not yet fully characterized the effect.) We reconstructed the 18 kbp deletion by replacement with a tetracycline resistance gene (Δ*18*::*tet*) (Figure 1C) and showed that the resultant strain had the expected phenotype [8]. The Δ*18*::*tet* mutation was introduced into wild-type cells by a standard *B. subtilis* transformation method (see Experimental Procedures). Transformants were selected on our standard L-form plates (nutrient agar [NA]/magnesium-sucrose-maleic acid [MSM]) containing tetracycline. The plates contain an osmoprotectant (sucrose) and an inhibitor of cell division (benzamide [17]) that inhibits the growth of walled cells, but not of L-forms. After ∼3–4 days at 30°C, small tetracycline- and benzamide-resistant colonies were visible (Figure 2A; the three large colonies marked by arrows contained rod-shaped walled cells and were presumably spontaneous tetracycline-resistant mutants or some kind of merodiploid recombinants). Phase-contrast microscopy of the small colonies revealed only L-form cells (Figure 2B). We confirmed the presence of the Δ*18*::*tet* mutation and deletion of the *murC* gene by PCR (see below). Consistent with our previous work [8], the newly selected L-forms were able to grow in liquid L-form medium (nutrient broth [NB]/MSM) in contrast to wild-type protoplasts not bearing the Δ*18*::*tet* mutation (Figure 2C). Certain types of L-forms are known to be able to regenerate cell wall and shape in the absence of selection pressure such as β-lactam antibiotics [5]. Proliferating L-forms induced by AccDA overproduction (*accDA*^∗^ *ispA*^∗^), and thus with an intact PG synthetic pathway, were indeed able to revert to the walled state, when spotted onto L-form plates without penicillin G. The left-hand spot in Figure 2D shows the emergence of dense colonies, which contained classical rod-shaped walled cells (Figure 2E, right panel). However, complete deletion of *murC*, e.g., by the Δ*18*::*tet* mutation, irreversibly blocks the PG precursor synthetic pathway and thus prevents regeneration of the cell wall (Figures 2D, right-hand spot with no dense growth, and 2F).

**Figure 3:**
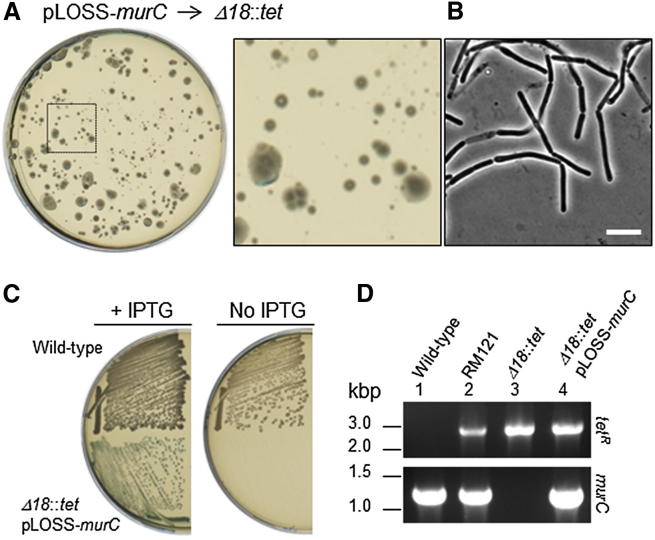
Cell Wall and Cell Shape Regeneration by the Restoration of PG Precursor Synthesis (A) Restoration of the cell wall and cell shape in Δ*18*::*tet* L-forms by reintroduction of the *murC* gene on a plasmid (pLOSS-*P*_*spac*_-*murC erm*^*R*^*lacZ*) using a PEG-dependent L-form transformation method (see text). Transformants were selected on an NA/MSM plate containing erythromycin and 2 mM IPTG. The plate was incubated for 3 days at 30°C after transformation. An enlarged image of the typical colonies (dashed square) is shown to the right. (B) Phase-contrast micrograph of cells from a typical colony on the transformation plate in (A). Scale bar represents 5 μm. (C) A transformant from the plate shown in (A) (Δ*18*::*tet* + pLOSS- *murC*) and a wild-type control strain were streaked on NA plates containing X-gal with or without IPTG. (D) PCR analysis for integration or removal of the Δ*18*::*tet* mutation and the *murC* gene in various strains: wild-type (lane 1), RM121(lane 2), Δ*18*::*tet* L-forms (lane 3), and Δ*18*::*tet* + pLOSS-*P*_*spac*_-*murC* (lane 4). RM121 (Δ*18*::*tet* + pLOSS-*P*_*spac*_-*murC*) was constructed in previous work [8] and was used as a control.

Having established a stable L-form strain incapable of cell wall synthesis, we wished to test whether the resumption of PG precursor synthesis would enable the regeneration of a PG cell wall and the restoration of rod-shaped cells. We attempted to introduce an isopropyl β-D-thiogalactoside (IPTG)-inducible ectopic copy of *murC* on a plasmid (pLOSS-*P*_*spac*_-*murC lacZ erm*^*R*^ [8]) into the Δ*18*::*tet* L-form strain by modifying an established polyethylene glycol (PEG)-mediated protoplast transformation method [18] (see details in Experimental Procedures). Transformants were selected on NA/MSM plates containing erythromycin and IPTG (for expression of the *murC* gene on the plasmid, *P*_*spac*_-*murC*) at 30°C. After ∼3–4 days, several erythromycin-resistant colonies appeared (Figure 3A). No colonies were seen on selective plates in controls lacking recipient L-forms, donor plasmid, or PEG treatment for transformation (data not shown). Phase-contrast microscopy revealed that these colonies were formed by walled cells with typical *B. subtilis* rod-shape morphology (Figure 3B). The colony-purified strain was able to grow on NA plates (without osmoprotectants) in the presence of IPTG, giving colonies that were blue in the presence of 5-bromo-4-chloro-3-indolyl-β-D-galactopyranoside (X-gal) due to expression of *lacZ* (β-galactosidase) from the pLOSS plasmid [19] (Figure 3C). We also confirmed the reintroduction of the *murC* gene into Δ*18*::*tet* L-forms by PCR (Figure 3D, lane 4). These results demonstrated that cells that have been propagated for a long period of time (at least 3 months; the proliferating L-form culture was maintained by diluting into fresh medium once per week) in a state in which they are unable to synthesize PG through loss of a key enzyme, are nevertheless able to regenerate a normal cell morphology on restoration of wall synthesis.

**Figure 4:**
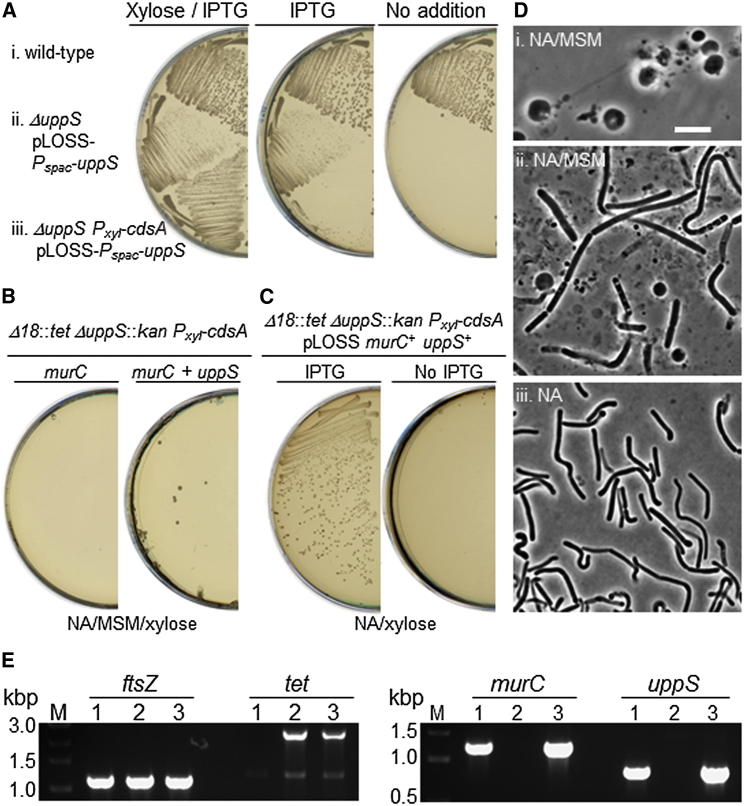
Regeneration of Rod-Shape Morphology by De Novo PG Synthesis in the Absence of an Existing Cell Wall Template (A) Effect of repression of *uppS* and *cdsA* on growth in the walled state. The following strains were cultured on NA plates with 1% xylose and 1 mM IPTG (left), 1 mM IPTG (middle), or without (right) at 30°C: wild-type (strain 168, i), Δ*uppS*::*kan* pLOSS-*P*_*spac*_-*uppS* (YK1888, ii), and Δ*uppS*::*kan P*_*xyl*_-*cdsA* pLOSS-*P*_*spac*_-*uppS* (YK1889, iii). (B) Regeneration of the cell wall in Δ*18*::*tet* Δ*uppS*::*kan P*_*xyl*_-*cdsA* L-forms by reintroduction of the *murC* gene (pLOSS-*P*_*spac*_-*murC erm*^*R*^*lacZ*, left) or *murC* and *uppS* genes (pLOSS-*P*_*spac*_-*murC P*_*uppS*_-*uppS erm*^*R*^*lacZ*, right) using a PEG-dependent L-form transformation method (see text). Transformants were selected on an NA/MSM plate containing erythromycin, 1% xylose, and 2 mM IPTG. Plates were incubated for ∼5–6 days at 30°C after transformation. (C) One of the transformants shown in (B) (right, Δ*18*::*tet* Δ*uppS*::*kan P*_*xyl*_-*cdsA* + pLOSS-*P*_*spac*_-*murC P*_*uppS*_-*uppS erm*^*R*^*lacZ*) was streaked on NA plates containing 1% xylose with (left) or without (right) 2 mM IPTG. (D) Phase-contrast micrograph of Δ*18*::*tet* Δ*uppS*::*kan P*_*xyl*_-*cdsA* L-forms on NA/MSM containing 1% xylose (i) and of cells (Δ*18*::*tet* Δ*uppS*::*kan P*_*xyl*_-*cdsA +* pLOSS-*P*_*spac*_-*murC P*_*uppS*_-*uppS erm*^*R*^*lacZ*) from a typical colony on the transformation plate (NA/MSM with 1% xylose and 2 mM IPTG) as shown in (B) (ii) or on NA plate containing xylose and IPTG as shown at left in (C) (iii). Scale bar represents 5 μm. (E) PCR analysis for integration or removal of the Δ*18*::*tet* mutation, the *murC* gene, and the *uppS* gene in various strains: YK1889 (Δ*uppS*::*kan P*_*xyl*_-*cdsA* pLOSS-*P*_*spac*_-*uppS*, lanes 1), YK1913 (Δ*18*::*tet* Δ*uppS*::*kan P*_*xyl*_-*cdsA*, lanes 2), and YK1925 (Δ*18*::*tet* Δ*uppS*::*kan P*_*xyl*_-*cdsA +* pLOSS-*P*_*spac*_-*murC P*_*uppS*_-*uppS erm*^*R*^*lacZ*, lanes 3). The *ftsZ* gene was also checked as a control.

To rule out the possibility of even a small amount of cell wall synthesis, we decided to build a host strain with an additional deletion in the *uppS* gene. The UppS product is a normally essential protein required for synthesis of undecaprenyl pyrophosphate (bactoprenol). Bactoprenol is an isoprenoid lipid carrier used for synthesis and export of precursors for both PG and wall teichoic acids (WTAs) (Figure 1A). We constructed a *uppS* deletion mutant in the presence of an IPTG-inducible copy of *uppS* carried on an unstable pLOSS plasmid (strain YK1888, Δ*uppS*::*kan* pLOSS-*P*_*spac*_-*uppS*). Growth of this strain was dependent on IPTG (Figure 4Aii), confirming that *uppS* is indeed essential for cell viability in normal walled cells. However, the growth defect was not fully restored in the presence of IPTG (Figure 4A, i and ii). We realized that *uppS* lies immediately upstream of *cdsA*, which is essential for membrane phospholipid synthesis. To avoid the polar effect on the *cdsA* expression, a xylose-inducible promoter (*P*_*xyl*_) was inserted in front of the *cdsA* gene to give strain YK1889 (Δ*uppS*::*kan P*_*xyl*_-*cdsA* pLOSS-*P*_*spac*_-*uppS*). The growth of YK1889 was similar to that of the wild-type in the presence of both IPTG and xylose, but no growth was seen in the absence of either IPTG or xylose, as expected (Figure 4Aiii). The Δ*18*::*tet* mutation was introduced into this strain (YK1889) to convert to the cells to L-forms; transformants were selected on NA/MSM L-form plates containing tetracycline, benzamide, and xylose. After ∼4–5 days, several tetracycline- and benzamide-resistant colonies appeared. Phase-contrast microscopy of the colonies revealed L-form cells (Figure 4Di), and we confirmed the presence of the Δ*18*::*tet* mutation, together with deletion of the *murC* and *uppS* genes by PCR (Figure 4E, lanes 2), showing that the strain had lost the *uppS* expression plasmid and therefore that L-forms do not require UppS protein. When this L-form strain was transformed with the pLOSS-*P*_*spac*_-*murC* plasmid, as described above, with selection on NA/MSM plates containing erythromycin, xylose, and IPTG, no colonies were seen on the selective plates (Figure 4B, left), consistent with expectation that the bactoprenol generated by *uppS* is necessary for the resumption of cell wall synthesis. In contrast, when a derivative plasmid carrying both the *murC* and *uppS* genes (pLOSS *murC*^+^ *uppS*^+^) was introduced into the L-form strain, several colonies appeared (Figure 4B, right). These colonies contained many walled cells with typical rod-shape morphology (Figure 4Dii). The colony-purified strain was able to grow with normal rod shape on NA plates (without osmoprotectants) in the presence of IPTG (and xylose, for the expression of *cdsA*) (Figures 4C, left, and 4Diii). PCR confirmed the reintroduction of the *murC* and *uppS* genes into Δ*18*::*tet* Δ*uppS*::*kan P*_*xyl*_-*cdsA* L-forms (Figure 4E, lanes 3).

In this report, we created a cell line in which PG synthesis is blocked by deletion of an essential gene, *murC*, in the PG wall synthetic pathway. We went on to generate an additional block in assembly of the precursors to both PG and WTAs, by deleting the *uppS* gene required for synthesis of the common lipid carrier bactoprenol. Although we cannot exclude the possibility that *B. subtilis* possesses a series of enzymes that can support wall polymer synthesis by an as yet undefined mechanism, it seems highly likely that the double blockade we generated would have abolished all significant wall synthesis. We then developed a transformation method for L-forms and returned the *murC* and *uppS* genes, enabling the resumption of cell wall synthesis. Ultimately, we showed that de novo PG synthesis is sufficient to regenerate the cell wall and restore a rod-shape morphology in *B. subtilis* in the absence of an existing cell wall template, conclusively excluding the need for a cell wall template in establishment of the rod shape of *B. subtilis*. What then are the mechanisms responsible for establishment and maintenance of cell shape? Several lines of evidence suggest that the actin-like MreB proteins play a central role in shape determination [10, 20–22]. Although molecular details of the function of these proteins remain elusive, the evidence that they regulate the synthesis of several key wall polymers during growth of the lateral wall is strong. Moreover, their ability to form extended linear filaments provides a means, at least in principle, of exerting long-range interactions on the cell wall synthetic machinery, leading to the control of gross cell geometry. It will be interesting to investigate the process whereby cell shape is reestablished, although the low frequency of this event precludes detailed analysis at present. Nevertheless, extension of the methods that we have developed for studying de novo cell wall synthesis promises to provide a powerful new means of studying the establishment of bacterial cell morphology.

## Experimental Procedures

### Bacterial Strains, Plasmids, Primers, and Growth Conditions

The bacterial strains, plasmid constructs, and primers for PCR analysis in this study are shown in Tables S1 and S2 available online. DNA manipulations were carried out using standard methods. Protoplasts were prepared as described previously [8]. Normal *B. subtilis* cells were grown on NA (Oxoid) and in Luria-Bertani broth. *B. subtilis* L-forms and protoplasts were grown in osmoprotective medium composed of 2× MSM (pH 7) (40 mM MgCl_2_, 1 M sucrose, and 40 mM maleic acid) mixed 1:1 with 2× NB (Oxoid) or 2× NA. Details of supplements, antibiotics, and microscopic imaging used for this study can be found in the Supplemental Information.

### Selection of Δ*18*::*tet* L-Forms

For selection of the Δ*18*::*tet* L-forms, we transformed chromosomal DNA of the strain RM121 (Δ*18*::*tet* pLOSS-*P*_*spac*_-*murC lacZ erm*^*R*^ [8]) into wild-type *B*. *subtilis* using standard methods [23]. Transformants were selected on L-form plates (NA/MSM) containing 30 μg/ml tetracycline and 1 μg/ml benzamide.

### Transformation Method for L-Forms

L-form transformation was carried out by modifying a PEG-dependent protoplast transformation method [18]. Proliferating L-form cultures (Δ*18*::*tet*) were diluted at 10^−3^ into fresh NB/MSM medium (10 ml) and incubated at 30°C until OD_600_ = ∼0.2 (2 days). The culture was centrifuged at 8,000 rpm for 10 min, and the L-forms were resuspended in 300 μl of NB/MSM medium and then mixed with 2 μg of *murC* expression plasmid. For L-form transformation, 150 μl of the L-form and plasmid mixture was transferred into 450 μl of MSM containing 40% PEG6000 (Sigma-Aldrich) and gently mixed. After 2 min, 1 ml of NB/MSM was added and mixed, and the cells were then centrifuged at 8,000 rpm for 10 min. The cell pellet was resuspended in 300 μl of NB/MSM and incubated for 120 min at 30°C. Finally, a 150 μl sample of the cell suspension was plated on NA/MSM plates containing erythromycin and IPTG. The plates were incubated at 30°C.

## Supplemental Information

Document S1. Tables S1 and S2 and Supplemental Experimental Procedures

Document S2. Article plus Supplemental Information
