# Supplementary material for: Bacterial Cell Morphogenesis Does Not Require a Preexisting Template Structure
Source: Curr Biol. 2014 Apr 14;24(8):863–7. doi: 10.1016/j.cub.2014.02.053 (PMC3989771; doi:10.1016/j.cub.2014.02.053)
Supplement: Document S1. Tables S1 and S2 and Supplemental Experimental Procedures [file mmc1.pdf]

Current Biology, Volume *24*

Supplemental Information

Bacterial Cell Morphogenesis Does Not Require a Preexisting Template Structure

Yoshikazu Kawai, Romain Mercier, and Jeff Errington

Supplemental Information

Supplemental Tables

Table S1. *Bacillus subtilis* strains and plasmid used in this study

Strain	Relevant genotype	Reference
168CA	*trpC2*	Lab. stock
RM121	168CA *Δ18*::*tet* pLOSS-P_{spac}-*murC erm lacZ*	Mercier et al., 2013 [S1]
YK1846[a]	168CA *Δ18*::*tet*	This study
RM84	168CA *xseB*::*Tn-kan*[b] *accDA**[c]	Mercier et al., 2013 [S1]
YK1848	168CA *Δ18*::*tet* pLOSS-P_{spac}-*murC erm lacZ*	This study
ΔuppS	168CA *ΔuppS*::*spc* pLOSS-P_{spac}-*uppS erm lacZ*	Patricia Domínguez-Cuevas, unpublished
YK1888	168CA *ΔuppS*::*kan* pLOSS-P_{spac}-*uppS erm lacZ*	This study
YK1889	YK1888 ΩP_{xyl}-*cdsA spc*	This study
YK1913[a]	168CA *Δ18*::*tet ΔuppS*::*kan* ΩP_{xyl}-*cdsA spc*	This study
YK1925	YK1913 pLOSS-P_{spac}-*murC* P_{uppS}-*uppS erm lacZ*	This study
Plasmid	Relevant genotype	Reference
pLOSS-*erm*-*murC*	*bla erm* P_{spac}-*murC lacZ*	Mercier et al., 2013 [S1]
pLOSS-*erm*-*murC*-*uppS*	*bla erm* P_{spac}-*murC* P_{uppS}-*uppS lacZ*	This study

tet, tetracyclin; *erm*, erythromycin; *spc*, specctinomycin; *kan*, kanamycin; *bla*, β-lactamase; *lacZ*, β-galactosidase

[a] These strains only grow as L-form.

[b] This mutation inhibits expression of *ispA* gene (Mercier et al., 2013) [S1].

[c] This mutation induces overexpression of *accDA* operon (Mercier et al., 2013) [S1].

Table S2. Primers used for PCR analysis

Primer	nucleotide sequence
Δ18::tet-F[a]	TTCGAACGGCCCGTCATTG
Δ18::tet-R[a]	TAAATCCAGGGCTTAGCCTG
murC-F[b]	AAAAGGGACCGGTATGAG
murC-R[b]	GACGTTTTCGTAGGCTCTC
uppS-F[c]	GTAGCTGTTCGTAACGTTCG
uppS-R[c]	TTTCATGTCCACCATCCTC
ftsZ-F[d]	ATGTTGGAGTTCGAAACAAAC
ftsZ-R[d]	TTAGCCGCGTTTATTACGG

[a] These primers set were used to amplify the tetracyclin gene to obtain *Δ18*::*tet* L-forms.

[b] These primers set were used to amplify the *murC* gene.

[c] These primers set were used to amplify the *uppS* gene.

[d] These primers set were used to amplify the *ftsZ* gene.

Supplemental Experimental Procedures

Growth conditions

Supplements, 2 or 1 mM IPTG and 0.004% X-gal were added as needed. When necessary, antibiotics were added to media at the following concentrations: 100 µg/ml ampicillin, 10 or 30 (for NA/MSM plates) µg/ml tetracycline, 1.5 µg/ml erythromycin, 5 µg/ml kanamycin and 50 µg/ml specctinomycin. 300 µg/ml PenG and/or Benzamide (1 µg/ml, FtsZ inhibitor 8J[S2]) was used for protoplast and L-form growth experiments to prevent the growth of walled cells.

Microscopic imaging

For phase contrast microscopy, cells from a liquid or solid culture were mounted on microscopic slides covered with a thin film of 1.2% agarose in MSM. The cells were imaged on a Zeiss Axiovert 200 M microscope equipped with a Sony Cool-Snap HQ cooled CCD camera. Pictures were prepared for publication using ImageJ and Adobe Photoshop.

Supplemental References

S1. Mercier, R., Kawai, Y., and Errington, J. (2013). Excess membrane synthesis drives a primitive mode of cell proliferation. Cell *152*, 997-1007.

S2. Adams, D.W., Wu, L.J., Czaplewski, L.G., and Errington, J. (2011). Multiple effects of benzamide antibiotics on FtsZ function. Molecular microbiology *80*, 68-84.
